# Objective measurement in Parkinson’s disease: a descriptive analysis of Parkinson’s symptom scores from a large population of patients across the world using the Personal KinetiGraph®

**DOI:** 10.1186/s40734-020-00087-6

**Published:** 2020-04-30

**Authors:** Rajesh Pahwa, Filip Bergquist, Malcolm Horne, Michael E. Minshall

**Affiliations:** 1 University of Kansas Medical Center, 3901 Rainbow Blvd, Kansas City, KS 66160 USA; 2 University of Gothenburg, Box 431 40530 Gothenburg, Sweden; 3 Florey Institute for Neuroscience and Mental Health, University of Melbourne, Parkville, Victoria Australia; 4 Centre for Clinical Neurosciences and Neurological Research, St Vincent’s Hospital Melbourne, Parkville, Fitzroy, Victoria 3010 Australia; 5 Certara Evidence & Access- 100 Overlook Center, Suite 101, Princeton, NJ 08540 USA

**Keywords:** Continuous objective measurement, Wearables, Parkinson’s disease, Motor symptoms, PKG

## Abstract

**Background:**

The Personal KinetiGraph® (PKG®) Movement Recording System provides continuous, objective, ambulatory movement data during routine daily activities and provides information on medication compliance, motor fluctuations, immobility, and tremor for patients with Parkinson’s disease (PD). Recent evidence has proposed targets for treatable symptoms. Indications for PKG vary by country and patient selection varies by physician.

**Methods:**

The analyses were based upon 27,834 complete and de-identified PKGs from January 2012 to August 2018 used globally for routine clinical care. Median scores for bradykinesia (BKS) and dyskinesia (DKS) as well as percent time with tremor (PTT) and percent time immobile (PTI) were included as well as proportions of PKGs above published PKG summary score target values (BKS > 25, DKS > 9, PTT > 1%, PTI > 10%). Two sub-analyses included subjects who had 2+ PKG records and scores above proposed BKS and DKS targets, respectively, on their first PKG. Median BKS and DKS scores for subsequent PKGs (1st, 2nd, etc.) were summarized and limited to those with 100+ subsequent PKGs for each data point.

**Results:**

Significant differences between countries were found for all 4 PKG parameter median scores (all *p < 0.0001*). Overall, 54% of BKS scores were > 25 and ranged from 46 to 61% by country. 10% of all DKS scores were > 9 and ranged from 5 to 15% by country. Sub-analysis for BKS showed global median BKS and DKS scores across subsequent PKGs for subjects who had 2+ PKGs and had BKS > 25 on their first PKG. There were significant changes in BKS from 1st to 2nd-6th PKGs *(all p < 0.0001).* Sub-analysis for DKS showed global median BKS & DKS scores across subsequent PKGs for subjects who had 2+ PKGs and had DKS > 9 on their first PKG. There were significant changes in DKS from 1st to 2nd and 3rd PKGs *(both p < 0.0001)*.

**Conclusions:**

This analysis shows that in every country evaluated a meaningful proportion of patients have sub-optimal PD motor symptoms and substantial variations exist across countries. Continuous objective measurement (COM) in routine care of PD enables identification and quantification of PD motor symptoms, which can be used to enhance clinical decision making, track symptoms over time and improve PD symptom scores. Thus, clinicians can use these PKG scores during routine clinical management to identify PD symptoms and work to move patients into a target range or a more controlled symptom state.

## Background

Parkinson’s disease (PD) is a progressive debilitating disease where continuous objective measurement (COM) can provide unique opportunities for improving symptom control. The global prevalence is 1–2 cases per 1000 population, or approximately 1–2% of those aged 60 years and older [1, 2]. PD affects many regions of the brain but damage to neural elements involved in dopaminergic neurotransmission [3] results in a number of motor and non- motor symptoms that respond to therapies with subsequent improvement in quality of life [4, 5]. The motor symptoms of PD bradykinesia, tremor, and rigidity are well known [6]. Bradykinesia is the cardinal diagnostic sign of PD and reflects impaired dopamine transmission. Thus measuring and treating bradykinesia can result in an improvement in other, dopamine responsive symptoms, including non-motor features of PD. [7–10] The main treatment of impaired dopamine transmission is levodopa [2]. Initially levodopa provides sustained benefit in treating PD symptoms but over time the duration of benefit of each dose becomes progressively shorter [11]. The clinical reemergence of bradykinesia and other motor and non-motor features are typically referred to as OFF periods or episodes. The improvement in symptoms between each dose is progressively shortened and can reach less than 2–3 h. In addition to this shortening of benefit from each dose, some people with PD (PwP) experience unreliability in the time to benefit and extent of benefit of each dose and even failure to gain any benefit from a dose (i.e., no ON period or dose failures). Collectively, variability in response to levodopa due to shortening and/or unpredictability of benefit are known as fluctuations. Involuntary movements known as dyskinesia frequently emerge after motor fluctuations begin and can be caused by excess, dysregulated dopaminergic transmission. After 5 years of disease [1, 3, 12, 13], approximately 50% of PwP can develop bradykinetic fluctuations and dyskinesia. Motor fluctuations and dyskinesia are the motor manifestations of reduced or excess (respectively) dopamine transmission, which also cause significant non-motor fluctuations [14].

Treatment of bradykinetic motor fluctuations [15] and dyskinesia depend on the PwP recognizing their presence and providing a history. However, PwP frequently under recognize wearing-off [16–18], bradykinesia and dyskinesia [19]. Many PwP have a new “perceived normal” where they may assume their uncontrolled symptoms are normal or uncorrectable [20]. Dyskinesia can be confused with tremor and bradykinesia can be attributed to tiredness rather than diminishing benefit from treatment. Additionally, PwP with declining cognitive function may also have difficulty attributing the emergence of symptoms to timing of medications. Symptom diaries are often used in clinical trials but the recording of signs and symptoms may be delayed to a more convenient time leading to potential recall bias [21–23]. In routine clinical practice, patient diaries are impractical and are not commonly used. For the patient (and caregiver), the challenge is in accurately and objectively recording and sharing this information effectively to optimize the decisions made based on the clinical visit. Thus, the management of levodopa dosing to alleviate or lessen ‘off’ time remains a challenge, since the reporting of ‘off’ time is subjective and varies from patient to patient.

Objective measurement offers an alternative to diaries that solves a dilemma for the patient and the clinician by capturing data during activities of daily living in the home environment. This not only relieves the burden for the patient on recalling events over the past few months, remembering to record the information but also in trying to articulate to their treating physician in a 15-min clinic visit. For the clinician it provides continuous objective information that can be easily interpreted in the nomenclature of PD. This can help to facilitate expeditated treatment to optimize patient outcomes.

Ambulatory continuous objective measurement (COM) technologies have been developed to overcome the problems encountered in self-reporting of dopamine responsive symptoms.

The Personal KinetiGraph® (PKG®) Movement Recording System is a new COM technology that provides scores of bradykinesia, dyskinesia, motor fluctuations, tremor, as well as immobility as a proxy for daytime sleepiness. One of the major advantages of a PKG device is that it is worn during routine activities and the PwP does not have to remember to perform specific tasks or record their activities during use. The PKG algorithms were designed to augment clinicians’ assessment of bradykinesia and dyskinesia and their assessment of the effects of therapy [24–30]. Having received regulatory clearance for PD patient use in Australia, Europe [31], and the United States [32], the PKG system is now used in routine patient care with over 35,000 PKGs having been performed worldwide [27].

The data collected by the PKG system is stored for research purposes in regional and global databases after de-identification. The de-identified PKG database contains measures of bradykinesia, dyskinesia, percent of time immobile, and percent of time in tremor, number of days worn by the PD patient, and basic demographic data including country of PD patient origin. When COM became available in other conditions, it inevitably led to the establishment of measurements that represent the boundary between subjects whose condition was adequately controlled and those in whom further therapeutic intervention would be in the patients’ best interest. These measurements thus become a therapeutic target and invite terminology such as “good control” of symptoms when target has been reached and “poor control” when it has not been attained. These targets are often based on several factors including physiology (i.e., values for the general population), evidence of improved quality of life or other outcome and economic/iatrogenic costs. These may shift over time as evidence changes. Targets for hypertension are a useful example. Recent publications discuss targets that have been set using a combination of expert opinion, normal physiology and empiric evidence with the expectation that research will further refine and modify these targets [33, 34]. Thus, it became possible to use these targets compared to scores in the PKG database to describe the proportion of people with bradykinesia and dyskinesia above these published target ranges. The usefulness of this provides clinicians with an objective measurement tool to more readily identify times of the day where PD treatments can be optimized to the benefit of PwP.

There is important evidence showing that increasing levels of bradykinesia, fluctuations and dyskinesia are related to health-related quality of life (HRQOL) and costs [4, 5, 35]. A recent study from the UK demonstrated that the average annual costs increased from £25,630 for those who spend less than 25% of waking hours in the off state compared to £62,147 for patients spending more than 75% of time in the off state [36]. A recent US study on the economic burden of PD estimated an incremental annual additional $24,439 to treat PD compared to matched subjects without PD in 2017 [37]. The same study showed a large percentage of PwP have experienced at least one OFF state in the past year (63.2%) [37].

## Methods

### Study objective

The major study objective was to describe objective symptom scores from the PKG system’s large, multinational, de-identified database and to relate these scores to published target ranges for patient reported outcomes in PD across different countries.

The PKG data was collected from PwP in countries across the world where the PKG device has been approved for use. Consistent with applicable privacy laws across the world, no identifiable protected health information (PHI) was extracted, accessed, or used during the course of the study. Pursuant to the USA Health Insurance Portability and Accountability Act (HIPAA) of 1996 with updated provisions [38], the EU General Data Protection Regulation (GDPR) of 2018 [39], and data privacy principles in Australia [40], our study used de-identified or anonymous data. Therefore, it does not require institutional review board (IRB) or ethics committee (EC) approval or waiver of authorization.

### The PKG® Technology and output

The PKG system design, measurement and output have previously been described in detail [33]. Accelerometry data is recorded by the PKG logger while worn on wrist for 6 days. The algorithms that are applied to this data produce scores of the severity of bradykinesia and dyskinesia over time [26]. The graphical and numerical output of the PKG is designed to be read and interpreted by the physician during a clinical evaluation [26]. PD patient status and progression can then be evaluated by comparing the graph and the scores of bradykinesia (BKS) and dyskinesia (DKS) with target ranges and relative to an age-matched group without PD [26, 27].

The PKG system consists of a data logger, a series of algorithms that produce data points every 2 min, and a series of graphs and scores that synthesize these data into a clinically useful format. The logger can be programmed to remind subjects to take their PD medications by delivering a vibration. Consumption of medications is acknowledged by the PwP through swiping the logger’s smart screen. The logger also has sensors to detect whether the device is being worn.

The algorithms were built using an expert system approach to model neurologists’ recognition of bradykinesia and dyskinesia on accelerometry data and to produce a bradykinesia score (BKS) and dyskinesia score (DKS) every 2 min. The PKG produces a graphical representation of the BKS and DKS collected every 2 min over an extended period (typically 6 days). Other scores include compliance with the reminders, percent of time with tremor [24], and times when the PKG device was not worn. The numerical output of the PKG device can be summarized in the following terms: median bradykinesia score (BKS) and median dyskinesia score (DKS), percent time with tremor (PTT), and percent time immobile (PTI). The PKG produces a BKS for each 2- min time period, and then selects the BKS at the midpoint (i.e., 50th percentile of all BKS for days worn) for all days the PKG was actually worn (usually 6 days) [9]. Bradykinesia was considered adequately treated if the BKS was < 25, which relates to a Unified Parkinson’s Disease Rating (UPDRS) scale of ~ 40 [26] and inadequately treated if the BKS was > 25 [9, 33, 34]. The PKG produces a DKS for each 2-min time period and then selects the score at the 50th percentile of DKS values for all days that the PKG was actually worn (usually 6 days). Dyskinesia was considered “controlled” if DKS < 9, which relates to an Abnormal Involuntary Movement Score (AIMS) of 10 [9, 33, 34]. The percent time immobile (PTI) was defined as the percentage of 2-min periods between 9 AM to 6 PM, where the movement data recorded by the PKG device was very low and correlated with the daytime sleep measured by polysomnography (PSG) and the Epworth Sleepiness Scale Scores (ESS) [29]. The percent time with tremor (PTT) was defined as the percentage of 2-min periods between 9 AM to 6 PM, that contained tremor [24]. Tremor is likely to be present if PTT score is > 1% [24].

### PKG database and statistical methodology

The Global PKG Database began in January of 2012 and by August of 2018, it contained 27,834 complete and de-identified PKGs from 21 countries where the device has received regulatory approval. Data from seven countries where more than 500 PKGs had been performed (referred to as the Top 7 countries) were analyzed and these constituted 94% (26,112/27,834) of the PKGs in the database. PKGs were excluded from this analysis if there was insufficient data to measure the scores required in this study. Specifically, PKGs were excluded if the logger was worn for less than 4 days, median BKS was equal to zero, there were negative FDS values or if one of the PKG values was missing. The data set includes any PKG loggers that had regulatory clearance including generation 1 and generation 2. The Kruskal-Wallis and paired t-tests were used for all analyses. Statistical significance was set at alpha < 0.05, and no adjustments were made for multiple comparisons. All analyses were performed using SAS 9.4 (Cary, NC – USA).

Median BKS and DKS scores, PTT and PTI were organized according to whether they were above or below published values for symptom control and are summarized in Table 1 [9, 23, 26, 27, 29, 33, 34]. Our study assessed change in scores over time focused on PwP from the Top 7 countries, stratified by BKS > 25 and DKS > 9 [9, 33, 34], respectively, on their first PKG and how both BKS and DKS scores changed with subsequent PKGs. A further interest was to examine how the scores of those subjects whose BKS and DKS initially were outside the target range changed with serial measurements. Data presentation was limited to those sites with *n* = 100 or more subsequent PKGs for each data point.

**Table 1:** Median PKG Symptom Scores and Proportion of Median Scores above Treatment Target Values

Top 7 PKG Countries; All (n, % of total PKGs)	Median BKS*	Median DKS*	Median PTT*	Median PTI*	As % of country’s PKGs			
					BKS > 25	DKS > 9	PTT > 1%	PTI > 10%
Australia (*n* = 8506, 31%)	25.8	1.8	1.2%	6.6%	51.8%	9.5%	53.3%	35.7%
UK (*n* = 5614, 20%)	26.5	1.8	1.3%	6.6%	55.5%	11.5%	54.7%	36.0%
USA (*n* = 4729, 17%)	27.5	1.2	1.9%	8.0%	61.2%	5.4%	65.1%	42.1%
Sweden (*n* = 2782, 10%)	25.0	2.3	1.1%	5.6%	48.0%	14.7%	50.8%	29.5%
Germany (*n* = 2070, 7%)	26.7	1.6	1.0%	6.9%	56.6%	8.5%	48.4%	36.3%
Netherlands (*n* = 1641, 6%)	26.0	1.9	1.0%	6.4%	52.4%	9.6%	48.0%	34.1%
France (*n* = 770, 3%)	24.6	2.8	0.8%	6.7%	46.4%	14.8%	45.1%	35.3%
All Countries (n = 27,834, 100%)	26.2	1.7	1.2%	6.8%	54.2%	9.9%	54.2%	36.6%

*p < 0.0001 for any difference among regions for all four objective symptom scores (BKS, DKS, PTT, and PTI)

*BKS* Bradykinesia Score, *DKS* dyskinesia score, *PTT* percent time in tremor, *PTI* percent time immobile

## Results

Table 1 summarizes PKG results from the top seven countries individually and from all 21 countries combined. Table 1 also shows the percentage of PwP from each country whose PKG scores were above threshold target value levels for BKS > 25, DKS > 9, PTT > 1% and PTI 10%. The median BKS in the US was higher (27.5) than in other countries (26.2) with a much higher proportion (61%) above target for bradykinesia. On the other hand, France (14.8%) and Sweden (14.7%) had the highest proportions of subjects with dyskinesia scores above target along with the lowest BKS scores. (DKS > 9%, or 9.9% for all countries, Table 1).

**Fig. 1:**
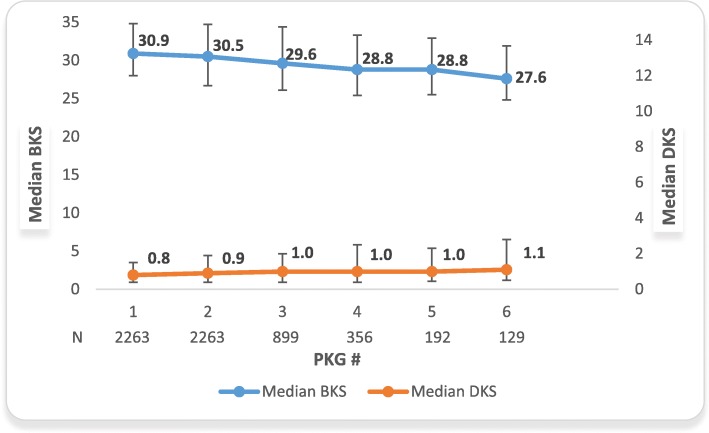
BKS Improvement of top 7 countries with > 2 PKGs and Baseline BKS > 25.5. *All *p < 0.0001* for comparing 1st to 2nd-6th BKS

The first sub-analysis (Fig. 1) included PD patients from the top seven PKG countries, with two or more PKG records, further stratified by BKS > 25.5 on their first PKG reading. The first sub-analysis was based on median scores of only those PD patients with serial PKGs (i.e., more than one PKG). There were statistically significant differences in BKS from 1st to 2nd through the 6th PKG readings in this stratified population (all *p < 0.0001*). The average time between each PKG order ranged from 23 to 42 days for the first 6 PKG readings. While BKS improved by 3.3 points (30.9 to 27.6 points), DKS increased by 0.3 points (0.8 to 1.1 points) suggesting that improvement in BKS by clinician use of more therapeutic agents did not adversely affect DKS which suggests no significant increase in side effects of abnormal movement for the PwP. sIn order to demonstrate stability of repeated PKG measures if PwP were “controlled” (BKS 18–25) at their first PKG, we report that serial PKG median BKS values were: PKG1, 21.90; PKG2, 22.30; PKG3, 23.00; PKG4, 22.80; PKG5, 22.20. The first median BKS > 25 was at the 11th PKG suggesting stability in repeated PKG measures in PwP who were initially considered within the target control range. We further assessed whether median BKS on the first PKG measurement differed for those PwP in the global database who had a single PKG, three PKGs, or more than 5 PKGs. The median BKS scores of the first PKG in the global database were 31.10, 30.65, and 30.63 for a single PKG, three PKGs, and five or more PKGs, respectively.

For BKS uncontrolled who got to a normal range after first PKG: 17.58% of population.

For DKS uncontrolled who got to a normal range after first PKG: 52.89% of population.

**Fig. 2:**
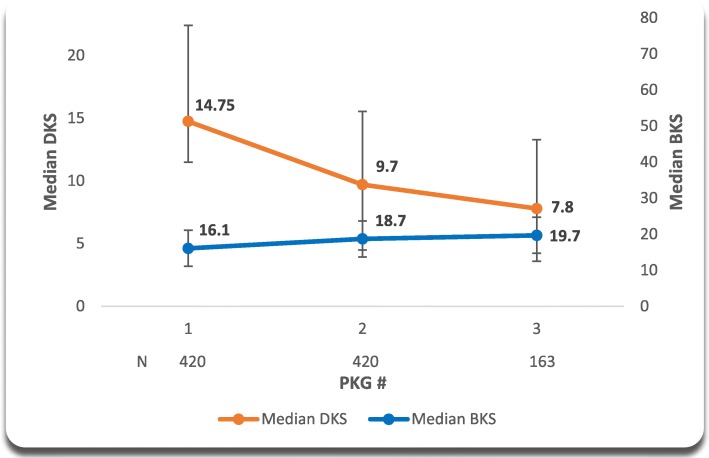
DKS Improvement of Top 7 countries with > 2 PKGs and Baseline DKS > 9.5. *All *p < 0.0001* for comparing 1st to 2nd and 3rd DKS

The second sub-analysis (Fig. 2) included PD patients from the top seven PKG countries, with two or more PKG records, stratified by DKS > 9.5 on their first PKG reading. There were statistically significant differences in DKS from 1st to 2nd and 3rd PKG readings in this stratified population (both *p < 0.0001*). For this sub-set, the DKS improved by 6.95 points (14.75 to 7.8 points), and the corresponding BKS increased by 3.8 points (16.1 to 19.7 points) suggesting that improvement in DKS representing more control of abnormal PD movement did not adversely affect BKS indicating a no impact to bradykinesia symptoms by increase in therapeutic agents to treat symptoms of dyskinesia.

## Discussion

Based on PKG measurements a significant proportion of the global database have sub-optimal motor symptom control and this proportion varies between countries. These scores most likely reflect the extent of sub-optimal control of motor symptoms in clinical management of PwP in those countries. In a study of a PD cohort that represented a community population, the median BKS of the population was 26.2 (which corresponds to a median UPDRS Motor III = 40) [9]. Only 17% in that study could not be further treated because of complications and 61% were treated with improvement in UPDRS I and II (and total) [9]. The use of PKG could reveal elevated and possibly treatable bradykinesia and dyskinesia symptoms. Previous studies suggest that most people whose scores are out of target could be treated to bring them within target range [9]. These studies suggest that being in target improves symptom control which may also be associated with improved health-related quality of life (HRQOL) in PwP. In this study, the observations suggest that PwP out of target range can be treated and thus see symptom improvement based on the data presented in Figs. 1 and 2. From a clinical perspective and following the treat to target concept, we note that (in Fig. 1) that the initial BKS of 30.9 is high. This score is out of the target range where a BKS of less than 26 would be normal. This indicates that a patient is likely experiencing bothersome symptoms of bradykinesia. The improvement in BKS by 3.3 points is a 19 percentage point reduction of healthy control BKS (median BKS of 18.6) or a reduction of 28% of the gap between healthy control median BKS and 30.9. The DKS of 0.8–1.1 is with the target range where a DKS greater than 9 is out of the target range. Additionally, an increase by 0.3 points for DKS is a normalization toward healthy control values where the median value is 4.3. From another perspective, this DKS increase from 0.8 to 1.1 is actually a 9% reduction of the gap towards the healthy control DKS value.

Additionally, when symptoms of bradykinesia represented by BKS or symptoms of dyskinesia represented by DKS were treated and a repeat PKG performed, no significant adverse impact on the opposing symptom was detected by significant change in PKG score. These PD symptoms could be identified, quantified, and enhance clinical decision making with use of the PKG system. Thus, by incorporating the use of objective measurement into routine clinical care of PwP, it offers clinicians a tool to more accurately collect and document times when the patient experiences PD symptoms throughout the day and from day to day in order to develop individualized treatment strategies throughout PD progression.

Motor fluctuations and dyskinesia have all been associated with reduced HRQoL and increased costs [34, 37]. There is some evidence that treating to objectively measured bradykinesia and dyskinesia target values will improve motor scores in under- and over-treated PwP, limit unnecessary medication use, and consequently improve short and long-term clinical outcomes [9, 33, 34]. Preliminary targets for any COM system needs to be established such that testing can be performed with incremental improvements made in the technology over time [33, 34]. Indications for use of COM systems with PD patients should be based on the need to discover unrecognized symptom changes resulting in therapeutic adjustments and improved communication between PD patients and health professionals [33, 34].

The PKGs in these countries mainly came from academic centers that specialize in the treatment of PD. Subjects are more likely to be attending these centers because of complex PD symptoms. The PKG is a tool that can be employed throughout the PD process to detect symptoms and to aim to bring PwP into a state of symptom control. Their first PKG may reflect this PD presentation complexity, which might subsequently be addressed by advanced therapies or other improvements in therapy. These could, for instance, account for increased levels of dyskinesia in Sweden and France. High levels of bradykinesia are less easily explained in this way and may in part reflect the difficult in discovering treatable bradykinesia (especially fluctuations) by history alone. Previous studies suggest that this may account for 30% of cases managed by movement disorder specialists. Country by country variations such as that observed in the US and UK are more likely to reflect differences in practice or care delivery. In population based samples [9], the scores may be more bradykinetic. There may be country differences but at the moment there is a selection bias that may skew results.

Our study uncovered county-specific variations in PKG assessments across several parameters.

The BKS > 25 indicating above threshold, ranged from a low of 46.4% in France to a high of 61.2% in the USA suggesting country-specific different patterns of pharmaceutical intervention, practice patterns, institutional practice, reimbursement and salary incentives, or overall physician experience (Table 1). Likewise, when DKS > 9 indicating above threshold, ranged from a low of 5.4% in the USA to a high of 14.8% in France suggesting the potential for under treatment in the USA while forcing PD treatment to the limit in countries such as France potentially increasing the probability of experiencing PD-related complications like dyskinesia symptoms as reflected in the PKG scores (Table 1). The PTT (> 1.0%) ranged from a low of 45.1% in France to a high of 65.1% in the USA (Table 1). The proportion of PKGs with tremor (PTI > 1.0) in each country closely reflected the proportion of PKGs with BKS > 25. This would be consistent with the implication that a PwP whose BKS > 25 would be more likely to be have tremor and be undertreated. The PTI (> 10%) ranged from a low of 29.5% in Sweden to a high of 42.1% in the USA (Table 1). A PTI > 10% may also indicate PwP who are more demented or untreatable rather than undertreated bradykinesia accounting for this difference.

### Limitations

Our analysis represents the PKG database for those patients who have actually used the device. PKG device users, to date, have largely been in specialized centers and in places where routine treatment of difficult PwP often occur and may skew our results. The study was also not specifically designed as a systematic collection of population representative data. The median number of PKG readings taken on the *n* = 27,834 patients in the database is 1.7, moreover, a large number of patients (*n* = 23,538) do not have a second PKG reading in the database which makes it difficult to assess longitudinal capabilities of the device. Secondly, the current BKS target value of > 25 is based on the current consensus as a reasonable treatment target may change over time and affect our results. Thirdly, Figs. 1 and 2 summarize PKG longitudinal data from the Top 7 countries for BKS and DKS scores that are based on diminishing number of patients for subsequent data points which may also introduce potential bias in representativeness of data for all PKG patients. However, these data represent an indication of the direction and magnitude of change in BKS and DKS scores for PD patients in a real-life setting across multiple countries. Other limitations include a lack of clinical data or patient outcomes collected in the database.

## Conclusions

COM in routine care of PD patients allows identification and quantification of PD motor symptoms which can be used in clinical decision making, tracking symptoms over time, and aiming to improve PD symptom scores. Based on this analysis, substantial regional variation in PD motor symptoms currently exist suggesting that improvements in patient management can be achieved in large groups of patients. A proportion of patients exist in each country that have uncontrolled PD motor symptoms with high levels of bradykinesia and daytime immobility reflected in more than half of the PKG scores globally. COM use in routine clinical care of PD enables identification and quantification of PD motor symptoms, which can then be used by clinicians to assess and track symptoms over time. This may begin to influence a shift in the PD management paradigm as COM and the use of target ranges becomes more imbedded in routine clinical practice.

## Data Availability

The datasets used and/or analyzed during the current study are available from the corresponding author on reasonable request.

## Abbreviations

AIMS: Abnormal involuntary movement scale
BKS: Bradykinesia score
COM: Continuous objective measurement
DKS: Dyskinesia score
ESS: Epworth sleepiness scale score
FDS: Fluctuation and dyskinesia score
GDPR: EU General data protection regulation
HIPAA: Health Insurance Portability and Accountability Act
HRQoL: Health-related quality of life measure
IRB: Institutional review board
PD: Parkinson’s disease
PKG: Personal KinetiGraph
PSG: Polysomnography
PTI: Percent time immobile
PTT: Percent time tremor
PwP: People with Parkinson’s Disease
